# Symptomatic corpus luteum hemorrhage in adolescent females with ITP

**DOI:** 10.1007/s00431-024-05560-0

**Published:** 2024-04-11

**Authors:** Alexander Yelak, Anat From, Oded Gilad, Dafna Brik Simon, Shiri Rubin, Miriam Cohen, Gil Amarilyo, Carina Levin, Doua Bakry, Shai Izraeli, Hannah Tamary, Joanne Yacobovich, Orna Steinberg-Shemer

**Affiliations:** 1 Department of Hematology-Oncology, Schneider Children’s Medical Center of Israel, Petach Tikva, Israel; 2 Gynecology Clinic for Girls and Adolescents, Schneider Children’s Medical Center of Israel, Petach Tikva, Israel; 3 Faculty of Medicine, Tel Aviv University, Tel Aviv, Israel; 4 Rheumatology Unit, Schneider Children’s Medical Center of Israel, Petach Tikva, Israel; 5 Ruth and Bruce Rappaport Faculty of Medicine, Technion - Israel Institute of Technology, Haifa, Israel; 6 Pediatric Hematology Unit and Research Laboratory, Emek Medical Center, Afula, Israel; 7 Department of Pediatrics, Ziv Medical Center, Safed, Israel; 8 Azrieli Faculty of Medicine, Safed Regional College, Safed, Israel; 9 Hematological Molecular Diagnostic Laboratory, Schneider Children’s Medical Center of Israel, Petach Tikva, Israel; 10 Pediatric Hematology Research Laboratory, Felsenstein Medical Research Center, Petach Tikva, Israel

**Keywords:** Thrombocytopenia, ITP, Corpus luteum hemorrhage, Bleeding

## Abstract

Patients with immune thrombocytopenia (ITP) usually present with minor mucocutaneous bleeding. Corpus luteum hemorrhage (CLH) is generally asymptomatic but may, rarely, lead to severe intraperitoneal bleeding, mostly in patients with coagulation disorders. CLH causing intraperitoneal bleeding has only been described in few individuals with ITP. The objective of this retrospective observational study was to assess the clinical course and incidence of symptomatic CLH in adolescent females with newly diagnosed or chronic ITP. Additionally, a comprehensive literature review was conducted to scrutinize cases of pediatric female patients with ITP, complicated by CLH. We identified three patients with ITP and hemoperitoneum secondary to CLH. They presented with acute abdominal pain, had severe thrombocytopenia (platelet counts below 20 × 10^9^/L), and required blood transfusions as well as ITP-directed therapy. All the patients were hemodynamically stable and did not require emergency surgical intervention.

*  Conclusion*: CLH could potentially pose a significant complication in the context of adolescent females with ITP, requiring a strong index of suspicion to direct expedient therapy.

**Table:** 

**What is Known:** *• Immune thrombocytopenia is typically associated with minor bleeding tendency.* *• Corpus luteum hemorrhage is generally asymptomatic; however, in women with bleeding disorders, it has the potential to result in substantial intra-abdominal bleeding.*	
**What is New:** *• Corpus luteum hemorrhage leading to intra-abdominal bleeding is a potential severe complication of immune thrombocytopenia in adolescent females.*

**What is Known:**

*• Immune thrombocytopenia is typically associated with minor bleeding tendency.*

*• Corpus luteum hemorrhage is generally asymptomatic; however, in women with bleeding disorders, it has the potential to result in substantial intra-abdominal bleeding.*

**What is New:**

*• Corpus luteum hemorrhage leading to intra-abdominal bleeding is a potential severe complication of immune thrombocytopenia in adolescent females.*

## Introduction

Immune thrombocytopenia (ITP) is the most common acquired bleeding disorder in children. Patients with ITP usually present with minor cutaneous bleeding including petechia and purpura. Some have mucosal bleeding, and a minority has severe manifestations, such as intracranial or gastrointestinal hemorrhage [1].

A corpus luteum (CL, yellow body) cyst is the natural result of ovulation and is hormonally active and progesterone-producing. These cysts may grow as a result of bleeding. In healthy women, bleeding into the CL is usually asymptomatic; therefore, the incidence of corpus luteum hemorrhage (CLH) remains unknown, often eluding detection by physicians. In cases where intra-abdominal bleeding occurs as a consequence of a ruptured CL, individuals may experience abdominal pain and tenderness, prompting diagnostic procedures that ultimately unveil the diagnosis of CLH-related hemoperitoneum. In such cases, patients are followed by their vital signs, physical examination, and hemoglobin levels to determine whether the bleeding is self-limiting or requires surgical intervention [2]. If needed, laparoscopy is performed, and in most cases, the bleeding can be controlled with cauterization or ovarian cystectomy; oophorectomy is usually not indicated.

Congenital or acquired coagulation factor deficiencies and anticoagulant treatment increase the risk for significant CLH [3–5]. Only a few cases of CLH leading to intraperitoneal hemorrhage have been described in adult women with primary ITP or ITP secondary to lupus erythematosus [6–9]. Conservative treatment, which includes transfusion of blood products, steroids, and intravenous immunoglobulins, has been sufficient in most cases.

In the adolescent female population, symptomatic CLH is rare; only three cases of adolescent females with ITP complicated with CLH and intraperitoneal hemorrhage have been described (summarized in Table 1) [10–12]. Similar to the adult published cases, all three presented with new-onset, severe abdominal pain, and had severe thrombocytopenia (< 20 × 10^9^/L) and severe anemia due to massive intra-abdominal bleeding from CLH. One patient underwent emergency splenectomy a week after presentation.

We aimed to shed light on the potential role of ITP as a risk factor for symptomatic CLH among adolescent females, and present three individuals with ITP and symptomatic CLH. Additionally, we studied all adolescent females with ITP diagnosed in our center over a 10-year period, to estimate the local prevalence of symptomatic CLH in this population.

**Table 1:** Clinical, laboratory, and treatment details of adolescent females with ITP and intra-abdominal hemorrhage secondary to CLH — published and presently documented cases

	**Reference**			**Patient no.**		
	Mäkipernaa and Nyman [10]	Yilmaz et al. [11]	Kaplan et al. [12]	1	2	3
Age, years	14	17	18	15	13	13
ITP disease stage	Newly diagnosed	Newly diagnosed	Newly diagnosed	Newly diagnosed	Newly diagnosed	Chronic
Platelets, ×10^9^/L	12	3	5	8	1	14
Hgb g/dL/HCT %	HCT 29	Hgb 5.6	Hgb 5.4	Hgb 6.6	Hgb 7.2	Hgb 7.4
Coagulation profile						
PT % (normal, 85–150)	NA	NA	NA	78	104	102
PTT, seconds (normal, 24–35)				30.4	25	38.1
Fibrinogen, mg/dL (normal, 200–530)				264	427	379
Treatment	Corticosteroids PC transfusion IVIg PLT transfusion	Corticosteroids PC transfusion IVIg PLT transfusion Splenectomy	PC transfusion IVIg Oral contraceptives	Corticosteroids PC transfusion IVIg Iron supplement	Corticosteroids PC transfusion IVIg Oral contraceptives Tranexamic acid	Corticosteroids PC transfusion IVIg Oral contraceptives Tranexamic acid

*HCT* hematocrit, *Hgb* hemoglobin, *PT* prothrombin time, *PTT* partial thromboplastin time, *PLT* platelet, *IVIg* intravenous immunoglobulin, *PC* packed cell, *NA* not available

## Methods

This retrospective observational study examined ITP patients with symptomatic CLH at the Schneider Children’s Medical Center of Israel since 2009. To determine local prevalence, we analyzed ITP cases (2009–2018), focusing on adolescent females as the reference population. The study was approved by our institutional review board.

## Results

We present the clinical spectrum of three adolescent females with ITP who had CLH and intraperitoneal bleeding, below and in Table 1.

### Case 1

A 15-year-old female presented with abdominal pain initiating on the day of admission. She had normal vital signs, but physical examination was notable for tenderness over the right lower abdomen. Her blood count revealed severe microcytic anemia (Hb 6.6 g/dL, normal, 12–16; MCV 70 fl., normal, 80–95) and severe thrombocytopenia (8 × 10^9^/L, normal, 150–450). Coagulation profile revealed a slightly prolonged prothrombin time (PT) of 78% (normal, 85–150) with a normal partial thromboplastin time (PTT) of 30.4 s (normal, 24–35 s), and fibrinogen concentration of 264 mg/dL (normal, 200–530 mg/dL). Blood smear showed pencil cells and paucity of platelets, while bone marrow (BM) aspiration was normal. She had positive anti-nuclear antibodies (1:160) and anti-DNA antibodies (38IU/mL, normal, <7), and low complement-4 levels (8 mg/dl, normal, 16–47) with normal complement-3 levels (90 mg/dL, normal, 86–186). Anti-phospholipid antibodies and lupus anticoagulant were negative. An abdominal ultrasound (US) demonstrated a right CLH of 13 cm. She was treated with packed cells (PC) followed by oral iron supplements. She received intravenous immunoglobulins (IVIg) and, due to a short response in platelet count, was also given a course of corticosteroids. Subsequently, hydroxychloroquine was initiated leading to normalization of the platelet counts. The patient eventually required a surgical intervention to remove a para-ovarian cyst 6 months after her initial presentation. She is now 3 years from diagnosis with ongoing hydroxychloroquine treatment and normal platelet counts.

### Case 2

A 13-year-old female, generally healthy, presented at another hospital with abdominal pain and epistaxis that initiated a day before admission. She had 10 × 10^9^/L platelets and a hemoglobin level of 11.9 g/dL. Her coagulation profile was within the normal range: PT 104%, PTT 25 s, and fibrinogen 427 mg/dL. Peripheral blood smear was normal except for a paucity of thrombocytes. She received a course of corticosteroids followed by IVIg. Due to a lack of response, she underwent a BM aspiration that was normal except for an increased number of megakaryocytes. During her hospitalization, her hemoglobin dropped to 7.2 g/dL and the platelet count remained low (13 × 10^9^/L). Physical examinations revealed diffuse cutaneous bleeding and petechia in the oral cavity, with notable findings of abdominal tenderness. Therefore, an US study was performed that showed a moderate amount of non-transparent abdominal fluid compatible with bleeding. She received a platelet transfusion and was transferred to our center. Her hemoglobin was 7 g/dL with 3.4% reticulocytes, and the platelet count was 1 × 10^9^/L. A left CLH and abdominal and pelvic fluid were identified through computed tomography imaging. She received PC, IVIg, and corticosteroids, and was also treated with tranexamic acid and oral contraceptives. The platelet count recovered gradually. The patient is now 3 years since diagnosis with normal platelet counts without treatment.

### Case 3

A 13-year-old female presented with a new finding of thrombocytopenia of 6 × 10^9^/L. In the 6 months prior to her admission, she noticed easy bruising on her extremities. On admission, the physical examination revealed a few hematomas on her lower extremities. She was treated with a course of corticosteroids followed by IVIg. Her platelet count stabilized at 20–40 × 10^9^/L, with no bleeding tendency, and she did not require additional treatment. Two years later, she presented with acute abdominal pain and her physical examination revealed lower abdominal tenderness. Her platelet count was 14 × 10^9^/L and hemoglobin level was 7.4 g/dL. Coagulation profile was within the normal range (PT 102% and fibrinogen concentration 379 mg/dL), except for a mildly prolonged PTT of 38.1 s. US studies revealed a moderate amount of pelvic hemorrhage; a right, well-defined 15-mm ovarian mass, compatible with CLH; and bilateral, small, simple follicular cysts of 6 mm. Overall, these findings conclusively supported the diagnosis of intraperitoneal bleeding secondary to CLH. She was treated with PC, IVIg, and tranexamic acid and discharged with oral contraceptives. A year later, she underwent a laparoscopic removal of endometrioma and a simple ovarian follicular cyst. Now, she is 4 years from diagnosis, receiving therapy with eltrombopag and oral contraceptives, and has normal platelet counts.

### Prevalence of symptomatic CLH

During 2009–2018, 256 patients in our center were diagnosed with ITP, of whom 31 were adolescent girls, aged 12–18 years. Hence, within our center, the occurrence rate of symptomatic CLH in adolescent girls with ITP was 9.7%.

## Discussion

ITP in children rarely causes severe bleeding [1]. Most patients present with cutaneous manifestations or mucosal bleeding, while life-threatening hemorrhage is rare. Intra-abdominal bleeding due to CLH is a severe complication that was previously reported in only a few adolescent females with ITP [10–12].

We presented three adolescent females with ITP who had CLH and intraperitoneal bleeding; two presented with CLH at the time of ITP diagnosis and the third had chronic ITP. The clinical presentation was similar in all cases, including an acute onset of abdominal pain. All three had platelet counts below 20 × 10^9^/L at the time of bleeding and hemoglobin levels that dropped to below 7.5 g/dL, requiring blood transfusion as well as treatment for ITP. None required hemodynamic support or emergency surgical intervention. Their coagulation profiles were normal to slightly disturbed, with normal fibrinogen levels. Therefore, disseminated intravascular coagulation was unlikely to have contributed to the bleeding manifestations or to the thrombocytopenia. Additionally, none of the patients had documented treatment with non-steroidal anti-inflammatory drugs or with other drugs affecting platelet function or number prior to their presentation.

Based on the occurrence of symptomatic CLH among adolescent female patients with ITP in our center, we suggest that CLH may be considered a severe complication of ITP, requiring a strong index of suspicion to direct therapy. Larger studies are needed to estimate the accurate prevalence of this complication among adolescent female patients with ITP.

## Data Availability

No datasets were generated or analyzed during the current study.

## Abbreviations

BM: Bone marrow
CLH: Corpus luteum hemorrhage
CL: Corpus luteum
ITP: Immune thrombocytopenia
IVIg: Intravenous immunoglobulins
PC: Packed cells
US: Ultrasound
